# Sedation methods for intra-articular corticosteroid injections in Juvenile Idiopathic Arthritis: a review

**DOI:** 10.1186/s12969-015-0021-0

**Published:** 2015-07-04

**Authors:** Amit Oren-Ziv, David Hoppenstein, Ayelet Shles, Yosef Uziel

**Affiliations:** Department of Pediatrics, Meir Medical Center, Kfar Saba, Israel; Department of Anesthesiology and Intensive Care, Meir Hospital, Tel Aviv University, Sackler School of Medicine, Kfar Saba, Israel; Pediatric Emergency Unit, Department of Pediatrics, Meir Medical Center, Kfar Saba, Israel; Pediatric Rheumatology Unit, Department of Pediatrics, Meir Medical Center, Tel-Aviv University, Sackler School of Medicine, Kfar Saba, 44281 Israel

**Keywords:** Juvenile idiopathic arthritis, Intra-articular corticosteroid injection, Sedation

## Abstract

Juvenile idiopathic arthritis (JIA) is the most common chronic rheumatic disease in children. Intra-articular corticosteroid injection (IASI), one of the cornerstones of treatment for this disease, is usually associated with anxiety and pain. IASI in JIA may be performed under general anesthesia, conscious sedation, or local anesthesia alone. Currently, there is no widely accepted standard of care regarding the sedation method for IASI. This review discusses the different methods of anesthesia and sedation in this procedure, emphasizing the advantages and shortcomings of each method.

## Introductory case 1

A 2 year old girl who has been diagnosed with oligoarticular juvenile idiopathic arthritis (JIA) is due for intra-articular corticosteroid injection (IASI) to her right knee. What is the recommended sedation technique for her?

## Introductory case 2

A 9 year old girl has a 4-year history of oligoarticular JIA. She has been taking methotrexate for a year and now has presented to the Rheumatology Clinic with exacerbation of left ankle arthritis. She had an IASI to the same joint a year ago. What would be the preferred sedation technique for her?

## Background

**Fig. 1:**
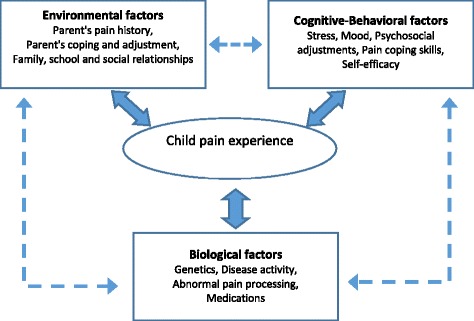
Factors influencing the child’s pain experience. Biological, cognitive behavioral and environmental factors interact to influence a child’s pain perception

JIA is the most common rheumatic disease of childhood [1]. Management of JIA includes a combination of pharmacological interventions, physical and occupational therapy, and psychosocial support [2]. Intra-articular corticosteroid injections (IASI) are one of the mainstays of treatment for children with JIA, particularly children with oligoarticular JIA [3]. It may be the only therapy needed for those patients, obviating the need for prolonged daily treatment. Studies have shown that as many as 70 % of patients with oligoarthritis do not have reactivation of disease in the injected joint for at least 1 year and 40 % for more than 2 years [4]. The most significant disadvantage of IASI is the pain and anxiety associated with the procedure. Various elements interact to influence the child’s pain experience. Among them are environmental, cognitive, behavioral and biological factors (Fig. 1).

Several studies in this field have shown that a child’s level of anxiety before a painful medical procedure is directly correlated with the amount of pain experienced [5, 6]. Since children with JIA frequently require repeated injections during their illness, extra care should be taken in making the injection procedure as painless and as stress free as possible, especially the first injection.

IASI in JIA may be performed under general anesthesia, conscious sedation, or local anesthesia alone. The choice of sedation or anesthesia should be tailored to the patient’s needs. The sedation can be administered by an anesthesiologist or by a non-anesthesiologist physician (e.g. nurse practitioner) who has undergone a specialized sedation course. Despite the frequent use of IASI in the management of JIA, there have been only few studies regarding the efficacy of different methods of anesthesia and sedation for this procedure [7]. Thus, there is currently no widely accepted standard of care in this field. This review discusses each of these methods.

### Local anesthesia

Local anesthesia used in IASI includes a variety of agents, such as eutectic lidocaine/prilocaine cream (EMLA) cream, subcutaneous lidocaine and ethyl chloride spray.

EMLA, a mixture of topical lidocaine and prilocaine, is a water emulsion cream applied to the skin under occlusive dressing 60 minutes prior to the procedure. It is widely used in young children for pain-related procedures [8]. Uziel et al. assessed its role in IASI and found it to have no statistically significant analgesic effect on pain associated with joint injections, when used alone [9]. The depth of EMLA analgesic effect has been shown to be approximately 3 mm after 60 minutes of skin application. The synovial membrane is usually deeper than 5 mm, which may explain the ineffectiveness of EMLA cream in achieving adequate analgesic effect for IASI. However, application of the cream has a placebo effect that has been reported in numerous studies [10]. The placebo effect by itself can alleviate some of the pain associated with painful procedures, including IASI.

Subcutaneous injection of lidocaine 2 % solution is commonly used for many procedures including joint aspiration and injection [7]. The administration of lidocaine can be painful, but buffering it with sodium bicarbonate has been shown to make its injection significantly less painful.

Another technique of local anesthesia for IASI is Lidocaine iontophoresis. It is a safe, non-invasive way of delivering topical lidocaine. Low-level electrical current drives the lidocaine into the skin [11]. The side-effects include erythema, tingling and itching at the application site. Iontophoresis may not be tolerated by all children due to the discomfort from the electrical current.

### Conscious sedation

The increased availability of short-acting sedatives along with accurate, noninvasive monitoring and improved sedation training programs have enabled effective and safe management of sedation and analgesia outside the operating room [12]. Several pharmacologic agents are being used in the setting of sedation for IASI.

### Nitrous oxide sedation

Nitrous oxide (N_2_O) is a volatile gas with analgesic, anxiolytic and sedative properties, when mixed with oxygen. It is an effective sedative and analgesic for mildly to moderately painful pediatric procedures. It has been reported that N_2_O can be safely administered up to 70 % concentration by nasal mask for pediatric procedural sedation, particularly for short (<15 minutes) procedures [13]. Studies have shown that N_2_O provides safe and effective analgesia for intra-articular injection in children [14, 15]. Common adverse effects reported include euphoria, nausea and vomiting, clinically insignificant hypoxia, dizziness, restlessness, and hallucinations. There have been no serious adverse effects reported with its use [16, 17].

Another advantage of N_2_O, in addition to its safety, is that the continuous flow method can be used in younger patients. It is also significantly less costly than general anesthesia.

Using N_2_O allows for a short hospital stay, avoids the risks associated with intravenous sedation and general anesthesia, and the procedure can usually be performed quickly after the need is recognized [18].

Disadvantages of N_2_O include the need for a mask or mouthpiece. Cleary et al. found that a mask was more likely to induce a state of anxiety and nausea in the patient [14]. Another disadvantage is the possibility of nasal breathing and dilution of the N_2_O component of the inhaled gas; hence, inadequate sedation effect. Experienced staff can overcome this problem.

### N_2_O combined with distraction techniques

Relaxation before and a comfortable atmosphere during sedation, make the procedure easier to perform. Cleary et al. reported relaxation techniques, including using a play specialist prior to the procedure, music and the use of laser light [18]. It is possible that these techniques explain their relative low visual analog scale for pain (VAS score, scored from 0 the lowest to 10 the highest) as compared to other similar studies (median VAS of 1, vs. mean of 2.1) [14, 15]. It is also possible that response to pain is culturally dependent and their lower scores are partially explained by the relatively calm British temperament.

The goal of distraction therapy is to shift the patient’s focus from the pain-inducing source. These methods have been shown to decrease pain levels and improve physiologic parameters, such as blood pressure and tachycardia.

Several studies showed that the presence of trained medical clowns significantly reduces preoperative anxiety in children [19–21].

In the past few years, distraction therapy has been encouraged for children undergoing painful procedures. Following the introduction of “Dream Doctors” (i.e. trained medical clowns) in our hospital, we added a medical clown as an integral part of the team performing IASI. We reported that active participation of a medical clown during IACS with nitrous oxide for JIA further decreases pain and stress, and induces a pleasant patient experience [22].

### Benzodiazepines

Benzodiazepines are commonly used for conscious sedation. The drug can be administered by oral, rectal, nasal, intramuscular and intravenous routes.

Midazolam is the most commonly used benzodiazepine for procedural sedation and analgesia. Time to peak effect for midazolam is brief with intravenous administration (2–3 min) and duration is short (45–60 min). Paradoxical reactions, characterized by inconsolable crying, combativeness, disorientation, agitation and restlessness, have been reported in 1–15 % of children receiving midazolam. They have also been reported with intramuscular, intranasal, and rectal administration of benzodiazepines [23].

This method of sedation requires insertion of an intravenous cannula, which is often a distressing procedure for the children. In addition, midazolam has no analgesic property, so for painful procedures it is commonly administered together with an opioid, a practice that may decrease the safety profile of midazolam due to the possibility of respiratory depression [24]. Due to the above, this method should only be used when adequate facilities for pediatric sedation and resuscitation are available. Midazolam can be used alone with local anesthesia as well in the cooperative child. Despite its disadvantages, it should be noted that when using standard precautions, the safety profile for midazolam is excellent and its anterograde amnestic effect is well recognized.

### Ketamine

Ketamine hydrochloride is a phencyclidine that binds to NMDA receptors and promotes intense analgesia, sedation and amnesia. It induces a trancelike state in which the patient is unaware, but not necessarily asleep. It can be administered by oral, intravenous and intramuscular routes.

With intravenous administration, peak concentration is achieved within 1 minute, allowing an immediate clinical effect. This route of administration allows deep levels of sedation to be achieved with good tolerance of extremely painful procedures.

Ketamine administration results in sympathetic stimulation and may increase heart rate and blood pressure, as well as intracranial pressure. Additional side effects include increased salivation, vomiting and rarely, apnea or laryngospasm. Despite the above, its safety in sedation among children has been largely reported [25].

### Propofol

Propofol is an ultra-short-acting potent sedative, hypnotic medication with no analgesic properties. Compared to other commonly used agents, propofol has the fastest onset of action and one of the shortest recovery times. Clinical effect is usually achieved within 30 seconds of administration. Adverse effects requiring routine intervention by appropriately trained providers occur in approximately 2-5 % of children undergoing propofol sedation and include respiratory obstruction, respiratory depression, apnea, and hypotension. Its main disadvantage as a drug used in procedural sedation is its narrow therapeutic index, i.e. its possibility of rapidly inducing anesthesia with loss of airway reflexes, upper airway obstruction and respiratory depression [26].

### Fentanyl

Sedative-hypnotic agents, such as propofol and midazolam do not have analgesic properties and need to be combined with other analgesic agents to provide effective sedation for painful procedures. Fentanyl is a synthetic opioid that provides analgesia for procedures with moderate to severe pain. Its rapid peak effect (within 5 minutes) and relatively short duration of action render it preferable for procedural sedation when compared to longer acting opioids, such as morphine [23]. Although typically administered parenterally, fentanyl also can be effective when used intranasally or via nebulizer.

Hypoxemia, respiratory depression, and apnea may occur when fentanyl is combined with other sedatives (e.g., propofol, midazolam). Chest wall and glottic rigidity are rare, but life-threatening adverse effects of fentanyl [27]. Because of the above, fentanyl should be given only by an anesthesiologist or by a non-anesthesiologist physician who has undergone a specialized sedation course and when adequate facilities for pediatric resuscitation are available.

### General anesthesia

Young children, or those requiring multiple joint injections, will require general anesthesia. Some pediatric rheumatology units routinely use general anesthesia for intra-articular injection. With modern anesthesia techniques, the children can often undergo this procedure in the anesthesia room under a short, general anesthetic, and all can be treated as day cases [18].

If the child is uncooperative in the pre-anesthetic phase, a number of effective non-traumatic measures may be implemented, such as distraction by a medical clown, parent or other members of the operating room team. Failing the above, oral midazolam syrup may be administered 30–45 minutes prior to induction of anesthesia [28].

The major advantage of general anesthesia is its rapid onset and offset of action. An example may include the inhalation by mask of 70 % N_2_O in oxygen for 2 minutes, followed by the introduction of a potent, volatile anesthetic agent like sevoflurane until consciousness is lost. This technique combines both agents in a synergistically, with the N_2_0 providing the analgesic component and sevoflurane providing the hypnotic component [29]. Anesthesia can be maintained with this mixture, while the child breaths spontaneously throughout the procedure. This is a good opportunity for painless venipuncture and procurement of necessary periodic blood tests. If there is no need for venipuncture, a general anesthetic of 3–5 minutes duration can be safely performed by a pediatric anesthesiologist without an IV line.

An alternative method is to have potential venipuncture sites pretreated with EMLA for a minimum of 45 minutes, give 2 minutes of N_2_O inhalation, perform painless venipuncture and then give intravenous propofol in sufficient quantity for the remainder of the anesthetic period. Again, the N_2_0 is given for analgesia during the procedure with the propofol providing the hypnosis. There is no need for supplemental analgesia, as these injections rarely have post-injection articular pain. From personal experience (DH), children awakening from these short anesthetics may be distressed by the unexpected appearance of an in situ IV cannula and cannot be consoled until its removal. Using both of the above methods, the child will typically emerge from anesthesia to consciousness at the end of the procedure and will be ready for home discharge in 90 minutes. Side effects may include nausea and vomiting and delirium upon emergence. Of interest, a small crossover study comparing propofol and sevoflurane on emergence agitation in repeat anesthesia for ocular examinations displayed significantly more agitation after sevoflurane (38 %) versus none in the propofol group [30]. For ultra-short general anesthetics, these effects appear to be rare.

## Conclusions

Intra-articular injection in JIA is a safe, rapidly effective treatment for synovitis. The procedure can be facilitated in an ambulatory care setting using local anesthesia with or without moderate sedation, or under general anesthesia in an operating room or office based environment.

As mentioned above, one of the disadvantages of IASI is the pain associated with the procedure. In one survey looking at the use of IASI for treatment of juvenile idiopathic arthritis, 43 % of respondents regarded procedure-related pain as a disadvantage of IASI therapy [31].

A number of individual differences affect children’s reports of painful events, including age, anxiety, temperament, pain response, and prior experience [32]. In 2 separate studies of medical procedures, children who had more distressing previous experiences were found to be more anxious and distressed than children who had primarily positive or neutral experiences with procedures [33, 34]. Since JIA is a chronic disease and many affected children will experience numerous injections during the course of their illness, much emphasis should be given in making the procedure as painless, stress free and even pleasant as possible.

In summary, few sedative methods are available to pediatric rheumatologists for IASI. There is no accepted standard of care regarding sedation, and each treating physician should feel comfortable and safe using the appropriate method. The decision should be taken after consulting pediatric anesthesia colleagues and taking into consideration the trained staff and the patient himself. Further studies are needed to determine the optimal method of sedation to minimize pain and anxiety associated with IASI.

## Back to the cases

### Case 1

This patient is very young and therefore will probably not cooperate with a painful procedure. It is very difficult for this age group to hold still**,** thus jeopardizing the accuracy of the injection. Therefore, in our opinion this patient should undergo the IASI under general anesthesia or moderate sedation. Since this girl will probably need repeated injections in the future, it is important to make the procedure as painless as possible for her in order to reduce anxiety before subsequent procedures. A positive family unit, child life specialist or medical clown may facilitate these as positive experiences.

### Case 2

In this case considering the patient’s age and experience with a former IASI, the preferred mode of sedation in our opinion would be N_2_O combined with a distraction technique, such as a medical clown. This way the patient will not have to face the stress of intravenous cannula insertion and will avoid the risks of benzodiazepine sedation. The presence of a medical clown will help make the procedure as pleasant as possible for her.
